# Feasibility and anteversion accuracy of a patient-specific instrument for femoral prosthesis implantation in total hip arthroplasty

**DOI:** 10.1186/s12938-023-01152-5

**Published:** 2023-09-13

**Authors:** Wei Zheng, Xuefeng Liu, Runhong Mei, Gaorong Deng, Zhipeng Li, Rongji Lin, Shui Xiong, Binghua Wu

**Affiliations:** 1 Department of Orthopaedics, The Fourth Affiliated Hospital, Nanchang University, Nanchang, 330003 China; 2 Department of Oral and Maxillofacial Surgery, The First Affiliated Hospital, Nanchang University, Nanchang, 330006 China

**Keywords:** Patient-specific instrument, Femoral anteversion, Total hip arthroplasty, 3D printing, Surgical guide

## Abstract

**Background:**

The aim of this study was to evaluate the precision and feasibility of patient-specific instruments (PSI) in total hip arthroplasty (THA) as compared to the traditional free-hand (FRH) approach.

**Methods:**

During the period of January 1, 2021 to December 31, 2022, a randomized allocation was used for patients receiving unilateral primary THA to either the PSI or conventional operation group. The placement and size of the PSI were specifically chosen to guide femoral neck resection and prosthesis implantation. The study analyzed component positions and evaluated radiographic and clinical outcomes in 30 patients who received PSI-assisted THAs and 30 patients who received FRH THAs. This study was registered at China Clinical Trial Registry (number: ChiCTR2300072325) on June 9th, 2023.

**Results:**

The use of PSI in THA resulted in significantly higher precision in achieving the desired component position as compared to the FRH approach. The PSI group showed significantly smaller absolute errors of femoral anteversion (*p* < 0.001). No significant differences were found in operation time, intra-operative blood loss, hospitalization duration, or time to walk after surgery.

**Conclusion:**

In conclusion, the application of patient-specific instruments in THA provides a simple and reliable solution to enhance the precision of femoral prosthesis placement with high accuracy and feasibility. This study highlights the potential benefits of using the PSI in THA.

## Introduction

Total hip arthroplasty (THA) is a widely recognized surgical procedure that offers an efficient and cost-effective solution for patients suffering from hip arthritis. However, despite its reputation as the “operation of the century,” modern THA remains imperfect, with significant risks associated with the procedure that may cause patient dissatisfaction [1–3]. Accurate positioning of the prosthesis is critical for a successful THA, as improper placement can result in complications such as accelerated wear, dislocation, impingement, leg length discrepancy (LLD), and other serious physical and financial burdens on the patient [4–10].

Numerous endeavors have been made to restore the hip joint's physiological anatomy in THA [11–13]. While computer navigation and robotics have improved implant positioning accuracy, their utility is limited by increased radiation exposure, longer surgical times, high costs, and the complexity of the surgical team [14–16]. Patient-specific instruments (PSI), a novel alternative to robotic and computer navigation, offer a simpler and more cost-effective solution for improving THA accuracy [17–20]. The benefits of PSI include ease of use, low cost, and high accuracy. However, creating a PSI for a single patient can take 2–3 days, in addition, there is a paucity of high-quality clinical data available on the use of PSI for femoral component implantation [21–23].

The progress of three-dimensional (3D) printing technology has exhibited promising outcomes in the realm of medical applications and holds potential in enhancing the efficiency of patient-specific instrumentation production. Therefore, the aim of this prospective study is to develop an innovative PSI system to ensure precise osteotomy and femoral component placement during total hip arthroplasty. Additionally, we aim to identify the accuracy of the PSI system and assess its clinical outcomes in comparison with the conventional approach.

## Results

A total of 80 patients underwent initial screening, out of which 60 were deemed eligible and subsequently randomized into the two study groups. The ICC score for interobserver was 0.965. Table 1 summarizes the demographic information of the patients, including age, gender, height, weight, and BMI, with no significant differences observed between the two groups (Table 1).

**Table 1:** Patient demographic date

	PSI (*N* = 30)	FRH (*N* = 30)	*p*-value
Age (years)	66.45 (30–96)	67.57 (35–90)	0.563^a^
Height (cm)	159.73 (148.00–173.00)	161.10 (140.00–180.00)	0.514^b^
Weight (kg)	54.38 (41.00–80.00)	57.74 (35.00–90.00)	0.244^b^
BMI (kg/m^2^)	21.21 (15.62–26.73)	22.06 (16.23–31.11)	0.500^a^
Sex (*n*, %)			
Man	13 (43.3%)	15 (50.0%)	0.608^a^
Woman	17 (56.7%)	15 (50.0%)	

*FRH:* free-hand group, *PSI:* patient-specific instrument group, *BMI:* body mass index

^a^Chi-squared test

^b^Student’s *t*-test; Values are *n* (%) or mean (ranges); *p*-value < 0.05 considered statistically significant

### Radiographic outcome

The planned femoral anteversion was 15.46° (range: 13.56–18.72°) in the patient-specific instruments (PSI) group, whereas in the FRH group, it was 16.50° (range: 13.61–21.97°) (*p* = 0.018). The study found that the actual anteversion in the PSI group was 20.02° (range: 15.75–27.80°), which was significantly different from the FRH group 23.86° (range: 20.31–30.64°) (*p* = 0.001). Furthermore, a considerable number of cases in the control group did not achieve the desired anteversion within 5°. The PSI group had 21 cases that met the desired anteversion within 5°, while the control group had only 13 cases (*p* = 0.037). For within 10°, the PSI group had 29 cases, while the control group had 25 cases (*p* = 0.085). In terms of absolute errors for stem anteversion, the PSI group had a mean of 4.56° (range: 0.60–11.9°), which was significantly smaller than that of the FRH group 7.38° (range: 0.30–15.00°) (*p* = 0.001) (Table 2).

**Table 2:** Comparison of preoperative and postoperative results

	PSI	FRH	*p*-value
Femoral component			
Planned anteversion	15.46 (13.56–18.72)	16.50 (13.61–21.97)	0.018^b^
Actual anteversion	20.02 (15.75–27.80)	23.86 (20.31–30.64)	< 0.001^b^
Absolute error anteversion	4.56 (0.60–11.9)	7.38 (0.30–15.00)	< 0.001^b^
Anteversion error > 5 (°)	9 (30.00%)	17 (56.67%)	0.037^a^
Anteversion error > 10 (°)	1 (3.3%)	5 (16.7%)	0.085^a^

*FRH:* free-hand group, *PSI:* patient-specific instrument group

^a^Chi-squared test

^b^Student’s *t*-test; Values are *n* (%) or mean (ranges);* p*-value < 0.05 considered statistically significant

### Clinical outcome

**Fig. 1:**
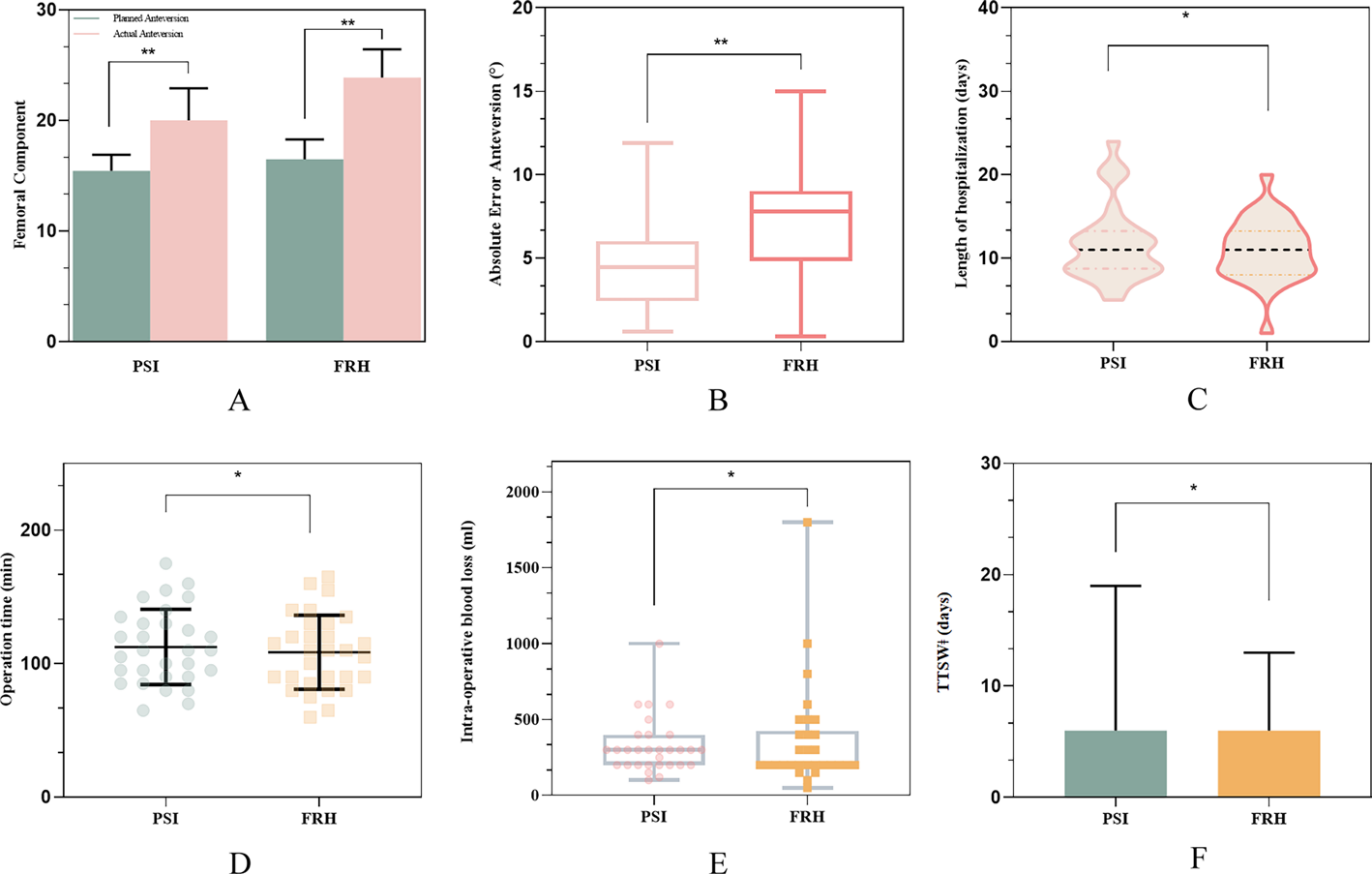
Postoperative result. **A** Error between preoperative plan and postoperative plan; **B** absolute error anteversion; **C** length of hospitalization; **D** intra-operative time; **E** intra-operative blood loss; **F** time to start walking on the ground after surgery; * the difference was not statistically significant (*p* > .05); ** the difference was statistically significant (*p* < .05)

The duration of surgery did not differ significantly between the PSI and FRH groups, with median operative times of 112.5 min (range 65–175 min) and 108.5 min (range 60–165 min), respectively (*p* = 0.581). Additionally, utilization of the PSI did not result in a significant increase in intra-operative blood loss compared to the FRH group, with median blood losses of 324 ml (range 100–1000 ml) and 361.67 ml (range 50–1800 ml), respectively (*p* = 0.598). The median length of hospitalization was 11.73 (range 5–24 days) in the PSI group and 10.97 (range 1–20 days) in the FRH group (*p* = 0.478) see Table 3. Additionally, no significant difference was observed in the time needed to start walking between the two groups following the surgical intervention (see Fig. 1). Moreover, neither of the groups experienced postoperative complications such as infection or dislocation.

**Table 3:** Intra-operative blood loss, total operation time, hospital stay and time to walk on the ground

	PSI	FRH	*p*-value ^a^
Length of hospitalization (days)	11.73 (5–24)	10.97 (1–20)	0.478
Operation time (min)	112.50 (65.00–175.00)	108.50 (60.00–165.00)	0.581
Intra-operative blood loss (ml)	324.00 (100.00–1000.00)	361.67 (50.00–1800.00)	0.598
TTSW (days)	7.00 (3.00–19.00)	6.37 (5.00–13.00)	0.364

*TTSW*: Time to start walking on the ground after surgery, *FRH*: free-hand group, *PSI*: patient-specific instrument group

^a^Student’s *t*-test; Values are mean (ranges). *p*-value < 0.05 considered statistically significant

## Discussion

In the real-world practice, the condition exhibits variability across different patients and posing challenge for surgeons in attaining the optimal component position. The main finding of this study was that utilizing the novel 3D-printed PSI improved accuracy of femoral anteversion achieved in comparison to the conventional free-hand procedure, regardless of patient characteristics. Although previous studies have primarily focused on the accuracy of acetabular implantation [8, 24–28], our research illuminates substantial advancements in both feasibility and precision in attaining the intended femoral prosthetic anteversion. In the PSI group, we achieved a more accurate anteversion compare to FRH group (*p* = 0.001). Also of note, our PSI technology resulted in a smaller absolute error of 4.56° than FRH group of 7.38° (*p* = 0.001). Similar to the previous study [29], in terms of surgical time, intraoperative blood loss, time to start walking on the ground after surgery and the hospital length of stay, no statistically significant difference was observed between PSI group and FRH group.

Component positioning is a crucial factor that affects both clinical outcomes and complication rates in hip arthroplasty [30–33]. Daisuke et al. [17] conducted a prospective clinical trial to investigate the accuracy of patient-specific navigation templates on THA. They found that the postoperative anteversion error was 6.1 ± 4.0° in the conventional group and 4.0 ± 3.5° in the PSI group. Zhang et al. [34] compared the results between the PSI group and the FRH group, found that the femoral prosthesis anteversion was 18.08° (range 12.53–21.91°) in the conventional group versus 16.76° (range 12.67–21.40°) in the PSI group (*p* < 0.005). In our study, PSI was more accurate in achieving actual anteversion than FRH group with small absolute error (*p* = 0.001). It is well accepted that in conventional procedure the precision and success of anteversion recovery mainly based on the surgeons' experience. Any error of pre- and intra-operative will affect the clinical outcome such as leg length, dislocation and implant survivorship. Based on our research, the PSI technology is a better choice for unexperienced surgeons to accurately achieve the targeted anteversion.

The results of the present study reveal that the operation time between PSI assisted group and FRH group was no significant difference. In contrast, previous work of Ferretti et al. [35], the team found that the mean surgical time in PSI group was 71.4 min longer than 60.4 min in FRH group (*p* < 0.05). Prolonged surgical time can cause adverse impact on patient clinical outcome such as increase the blood loss and infection rate. In the study conducted by Wang et al. [36], they retrospectively reviewed 17,342 patients who underwent total joint arthroplasty and found that each 20 min surgical time increase was associated with nearly 25% increased risk of total joint arthroplasty. Similarly, Anis et al. [37] followed up 11,840 patients with primary total knee arthroplasty for 2 years and found that longer operation time was an independent predisposing factor for prosthetic joint infection. We postulated that the variations in surgical time were due to factors such as the time for PSI location, time to fixed pin placement, and learning curve. In addition, the discrepancy of team collaboration and patient demographics may also exert significantly impact on the ultimate surgical time. We believed that as the surgical experience increases, there will be a steady reduction in the operation time when using PSI.

An efficient health care system aims to maximize health benefits with minimal resources. Indeed, the utilization of 3D printing technology may initially be perceived as financially demanding, given the customary expenses associated with PSI, encompassing preoperative CT scans, material consumption, and the intricate design of custom instruments. Notwithstanding these visible initial expenditures, the integration of 3D printing technology emerges as a wise investment, ultimately yielding substantial cost efficiencies over the extended term through the mitigation of various economically burdensome perioperative occurrences. Inappropriate femoral anteversion restoration can increase the revision rate due to the error of limb lengthening and decrease in hip offset [38]. According to the study of Kevin et al. [39] the mean hospitalization costs were (mean ± SD, USD 24,697 ± USD 40,489) for revision THA. David et al. [40] conducted that 3D printed PSI in patients’ operative care provides considerable value to health systems by reducing operating room costs. Based on our study, the cost of patient specific instrument system was less than $100. Overall, the PSI is a cost-effective assistance system.

The present study is not without limitations. First, our observation encompassed a limited scope of clinical outcomes, overlooking major complications such as dislocation, aseptic loosening, periprosthetic joint infection, and revision. To comprehensively assess the impact of increased accuracy facilitated by PSI-assisted THA on patient long-term outcomes, more extensive investigations with extended follow-up durations are imperative. Second, our focus was predominantly on comparing the accuracy of PSI with the conventional free-hand technique, neglecting a comparison with other technological approaches such as robots or navigations. Therefore, future investigations that encompass comparisons between PSI, robot, and navigation would undoubtedly enhance the understanding of this novel PSI technique. Third, the sample size of this study was modest. To further refine the viability and precision of the PSI instrument, larger-scale studies with prolonged follow-up periods are indispensable.

## Conclusion

Compared to conventional free-hand total hip arthroplasty, patient-specific instruments assisted THA was shown to result in more accurate in the variable anteversion of the femoral component of THAs. Our study suggests that PSI is a useful and feasible alternative for surgeons who seek to achieve precise surgical outcomes. Moreover, PSI has the potential to lower the incidence of complications and enhance implant survival.

## Material and methods

### Study design

All patients provided their informed consent prior to participating in this study. From January 1st 2021 to December 31th 2022, 80 cases undergoing primary total hip arthroplasty were prospectively screened for inclusion. Eligibility criteria included patients with unilateral hip disease, Crowe type I and II dysplasia of the hip, femur neck fracture, and who had received computed tomography (CT) screening before and after surgery. In addition, patients had to provide written informed consent to participate. Patients who had undergone prior hip surgery, had bilateral hip osteoarthritis, Crowe type III and IV dysplasia of the hip, or refused to participate were excluded from the trial. In this study, one patient was randomly assigned to the PSI group every two weeks, while the remaining patients underwent the FRH procedure. The randomization process was carried out by a third-party investigator who was not involved in the surgical procedure, and the patient’s name was selected from an opaque envelope. The surgeon was blinded to the assigned intervention until the start of the procedure, while the patients and research fellows remained blinded throughout the trial.

### Demographic characteristics

**Fig. 2:**
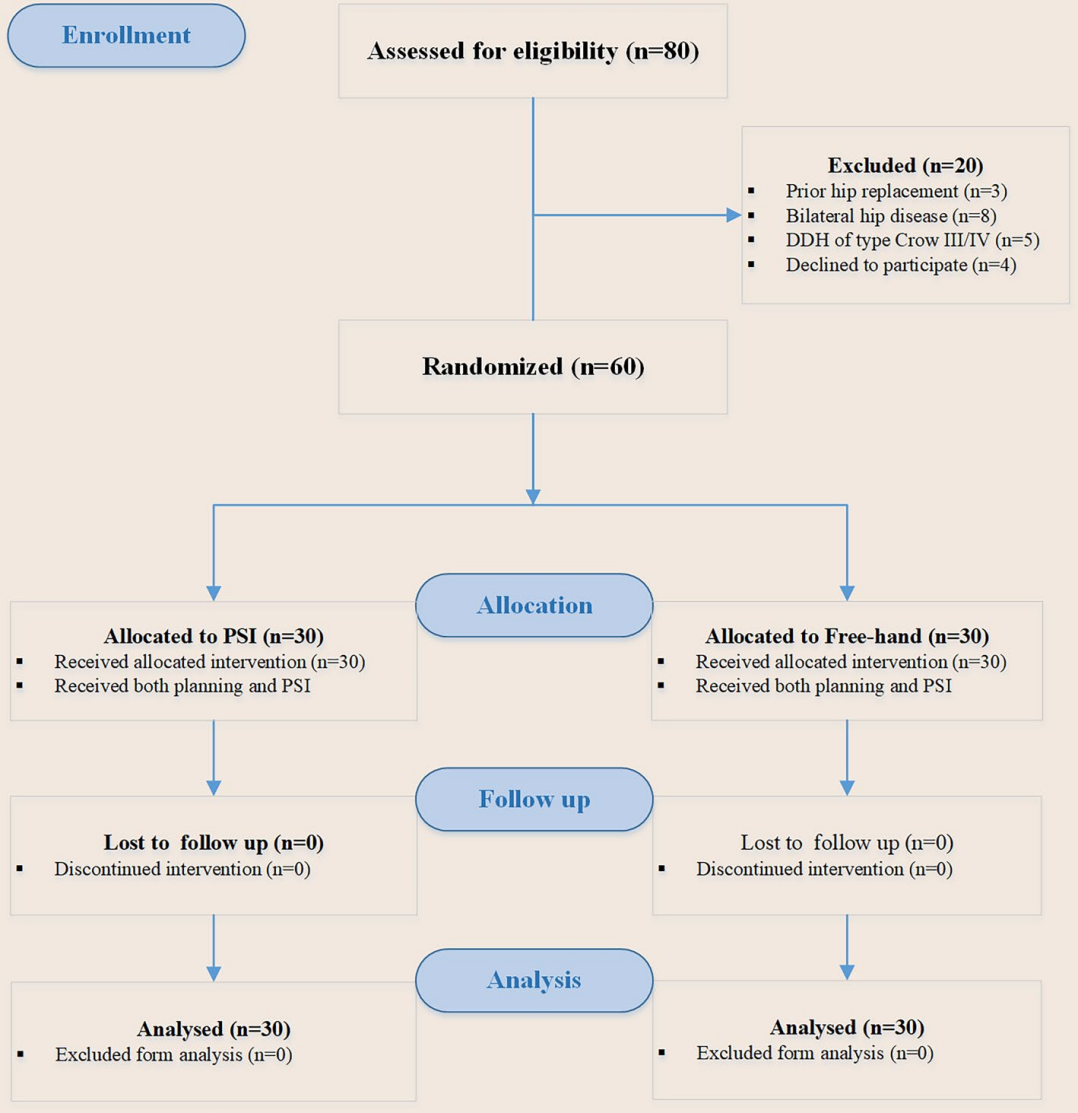
Work flow of this research

This investigation involved a cohort of 60 patients, with 30 receiving PSI-assisted THA and the remaining 30 undergoing FRH THA. The two groups were matched in terms of age, gender, height, weight, and body mass index (Table 1). The primary diagnoses in the PSI group included 5 cases of osteoarthritis, 11 cases of osteonecrosis, 4 cases of hip developmental dysplasia (Crowe I), and 10 cases of femoral neck fracture. The main diagnoses in the FRH group were three cases of osteoarthritis, nine cases of osteonecrosis, fifteen cases of femoral neck fracture, and three cases of hip developmental dysplasia (Crowe I) (see Fig. 2). There were no reported occurrences of complications, such as periprosthetic joint infection, intraoperative fracture, periprosthetic fracture, or dislocation in either group. The PSI group had a mean hospital stay of 11.73 days (range: 5–24 days), while the FRH group had a mean hospital stay of 10.97 days (range: 1–20 days). There was no statistically significant difference between the two groups (*p* = 0.478).

### Preoperative planning

**Fig. 3:**
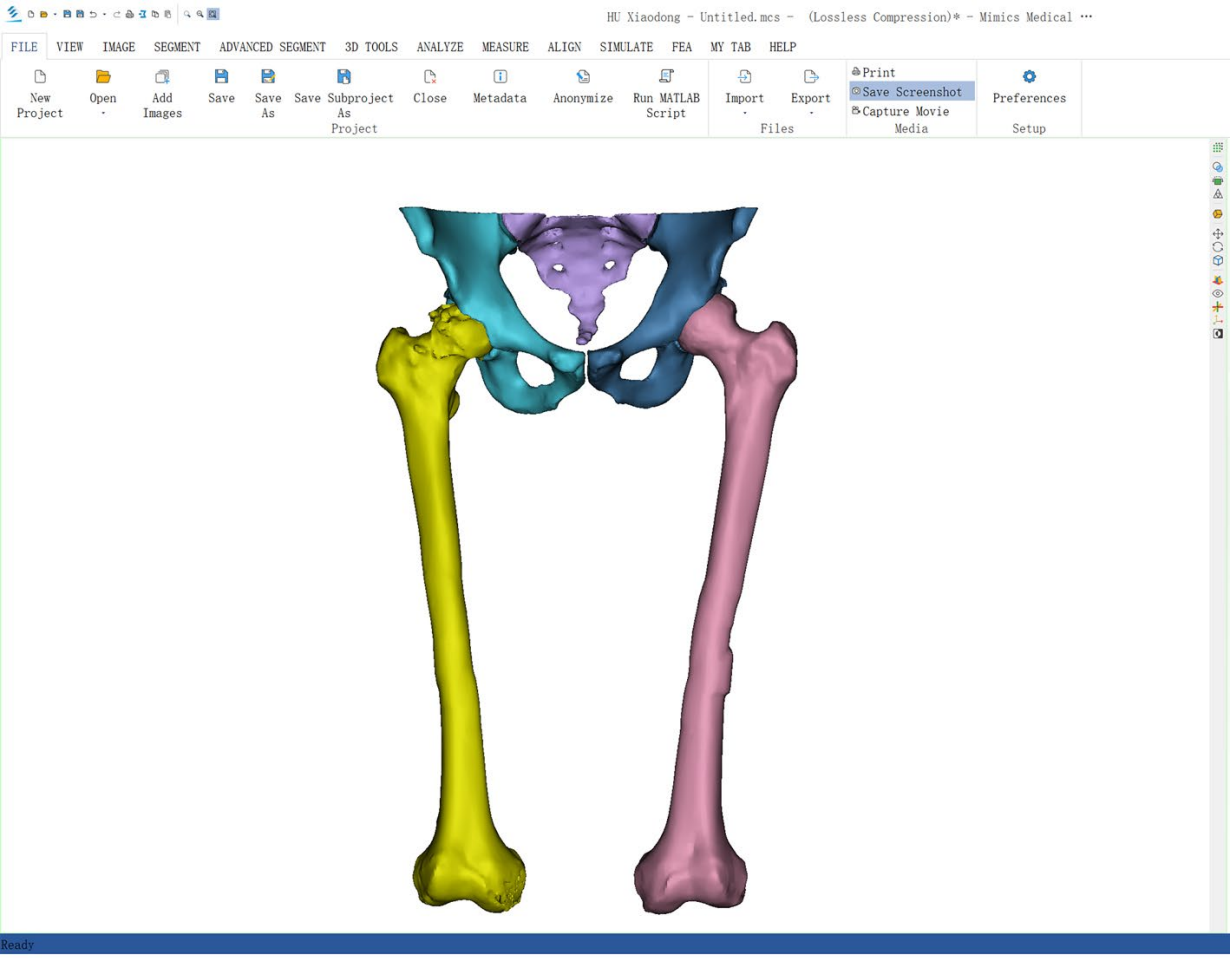
Building model by threshold difference and manual segmentation

**Fig. 4:**
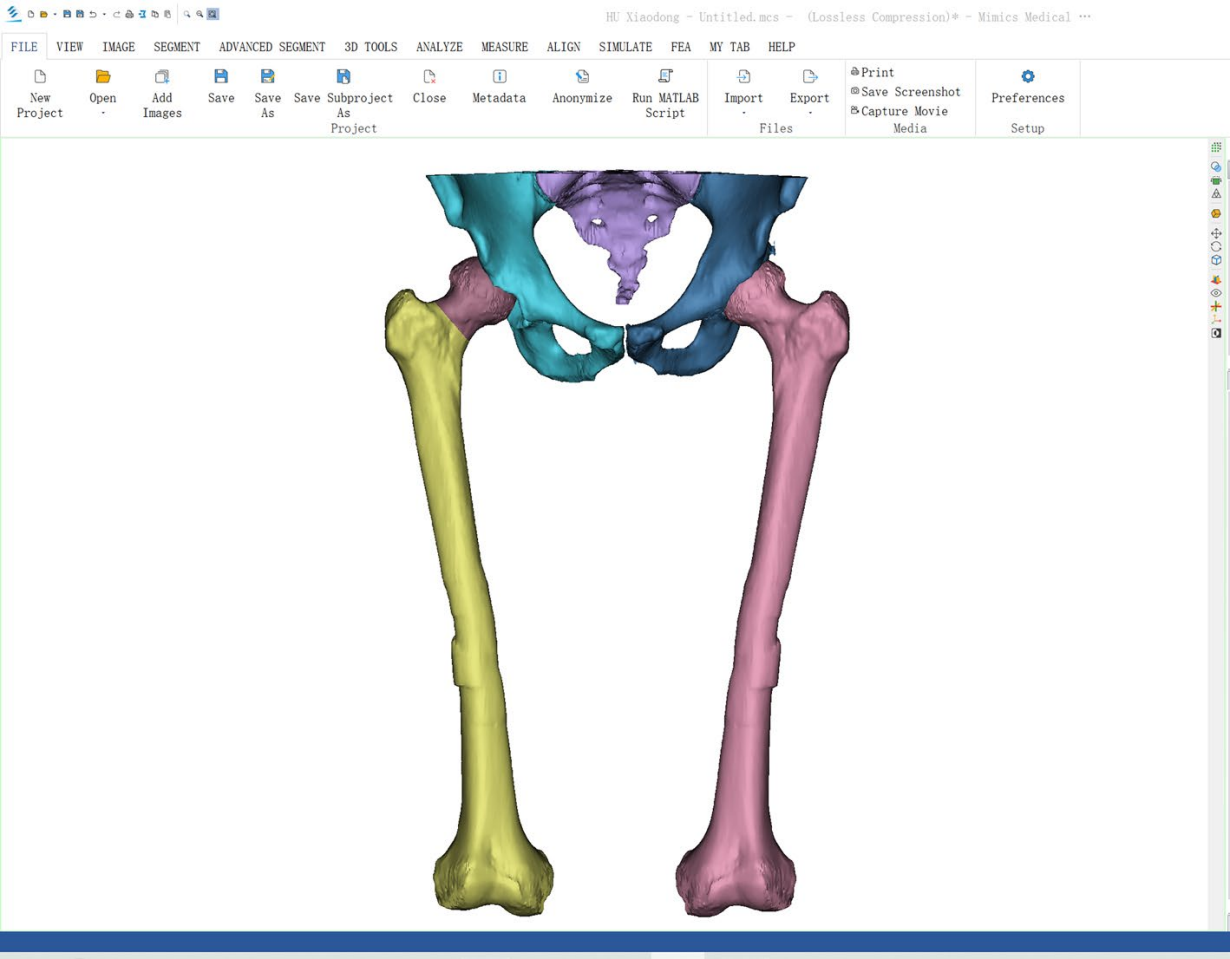
Mirrored contralateral hip joint

All patients underwent traditional two-dimensional template selection to determine the appropriate prosthesis. Preoperative computed tomography (CT) scans of the pelvis and femur were conducted on a Siemens scanner with 120 kV, 350 mA, and a layer spacing of less than 1 mm. Using threshold differentiation and manual segmentation, the CT images were imported into Mimics 21.0 software (Materialise, Belgium) to create separate models of the pelvis, healthy femur, injured femoral head, and proximal shaft (see Fig. 3). The desired femoral head position was determined by fitting the articular surface after virtual fracture reduction or registering the mirror image of the healthy model (see Fig. 4). The femoral stem prosthesis model was imported into the software, and the surgeon adjusted the posture of the prosthetic stem in an alternate view by referencing the target femoral head's location.

### Patient specific instrument

**Fig. 5:**
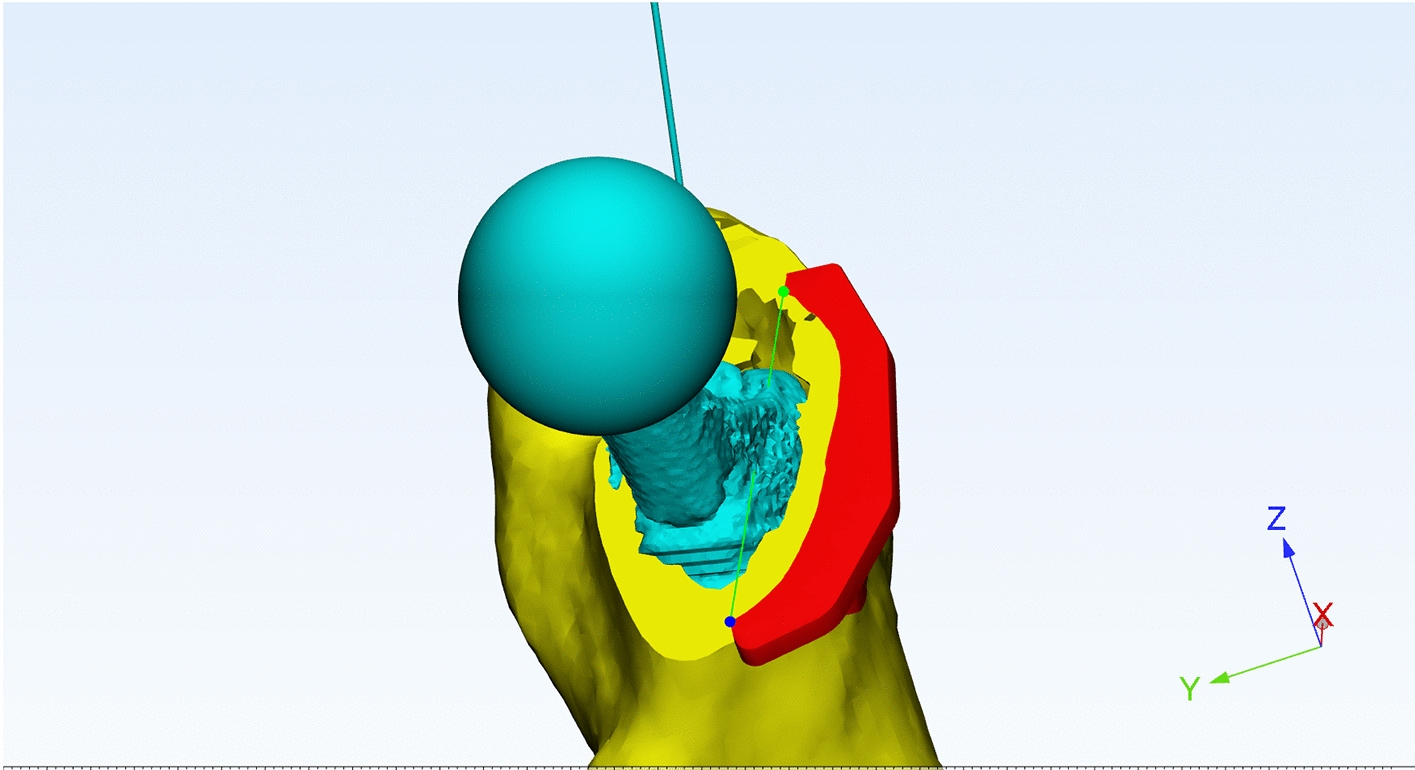
Determine the anatomic femoral anteversion

**Fig. 6:**
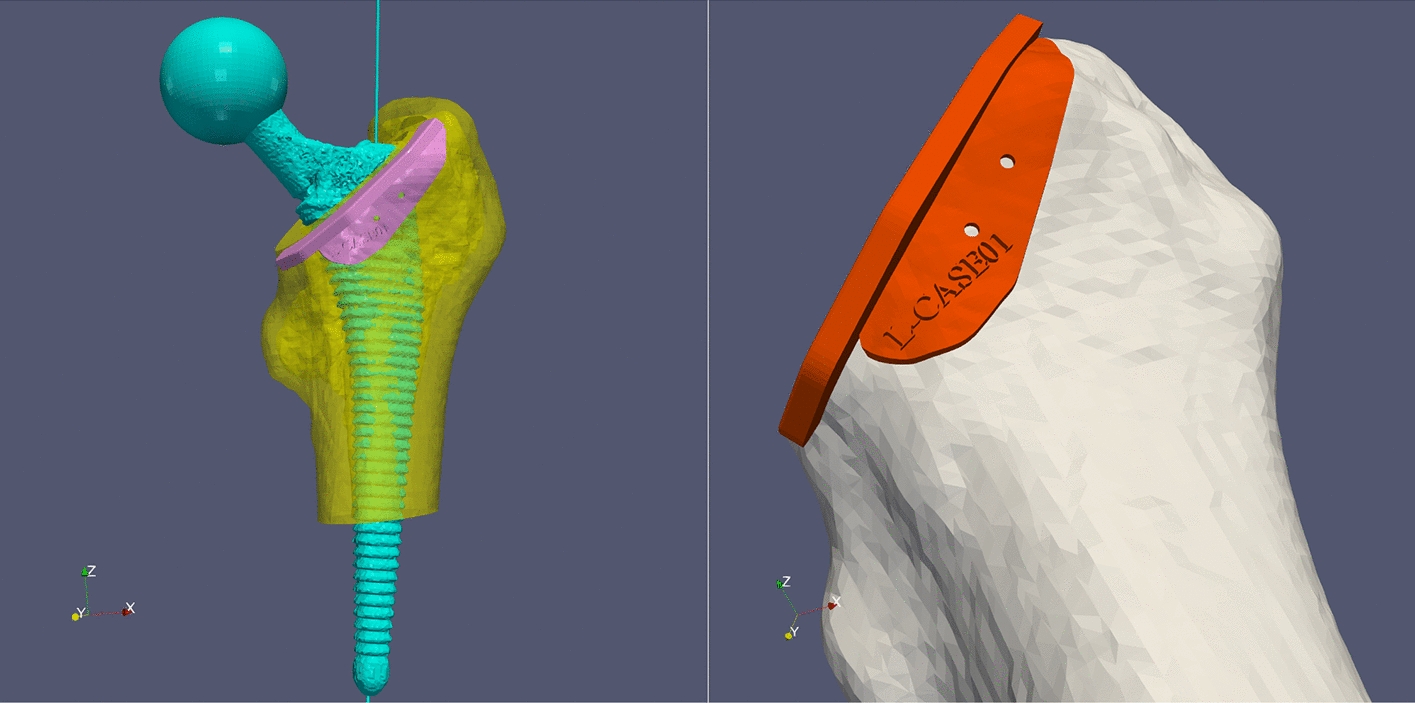
Design of PSI

All patients underwent CT scans of the bilateral femurs using DICOM-formatted raw data, which were processed with a commercially available 3D rendering software package (Mimics, Materialise, Ann Arbor, MI) to create precise 1:1 ratio 3D models. Soft tissue artifacts were removed to expose the bony femoral surfaces. Following the evaluation and assessment of each PSI design by two orthopaedic surgeons, 3D models of the acetabulum and proximal femur were produced, together with surgical guidelines based on the preoperative plan. The native femoral anteversion and anatomic axis of the femur were identified by mirroring the contralateral hip joint. The PSI, which had three supporting feet that matched the patient's unique anatomy and a plane with two guide margins showing the anatomic femoral anteversion (see Fig. 5), was then exported in STL format to Materialise Magics 24.0 software (Leuven, Belgium). The resulting data were used to create a resinous PSI (accuracy, 0.1 mm; material, photosensitive resin) with Materialise Magics 24.0 software (see Fig. 6). The average time between CT data acquisition and PSI creation using 3D rendering software was less than 24 h. Prior to use in surgery, the PSI components were sterilized with low-temperature plasma.

### Surgical technique and preoperative management

**Fig. 7:**
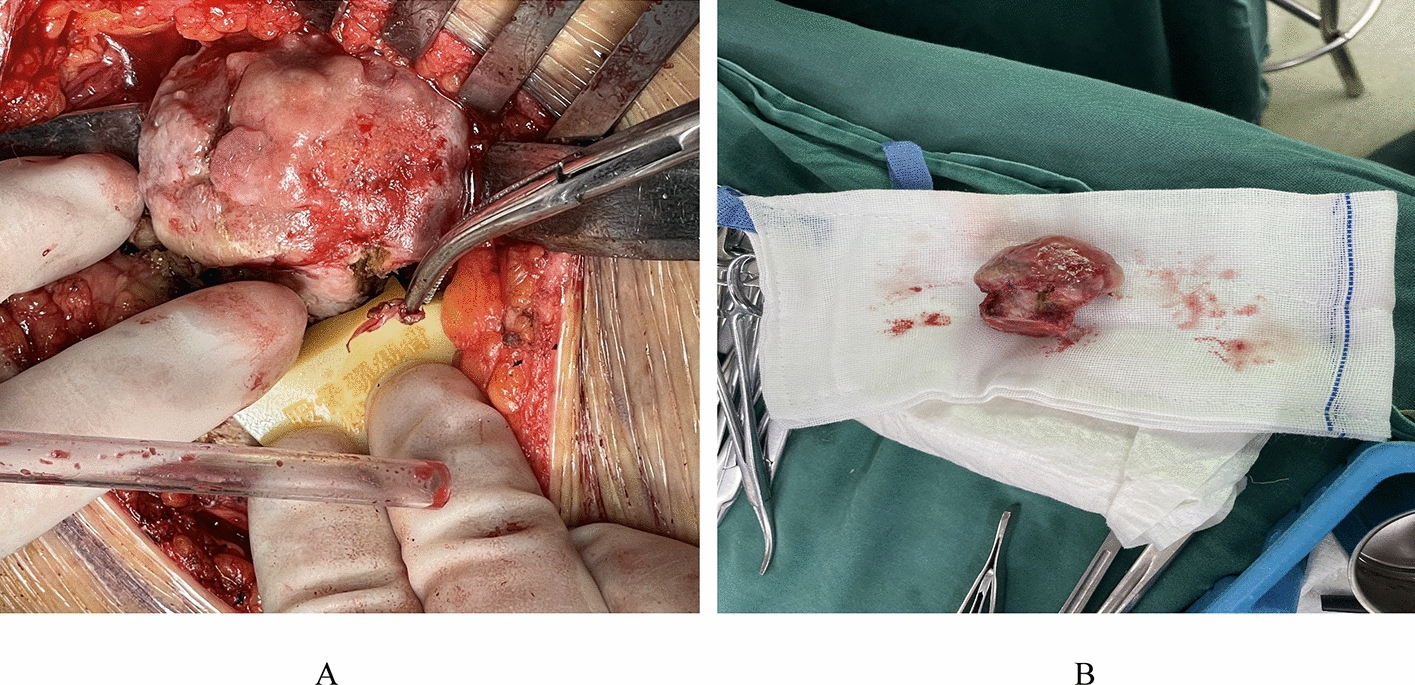
Intro-operative use of PSI. **A** Intro-operative osteotomy, **B** resected femoral head

In this study, all patients underwent total hip arthroplasty performed by a single surgeon who had no prior experience with patient-specific instruments used in this investigation. Upon administration of general anesthesia, the patient was positioned appropriately and a skin incision was made. The surgical team proceeded with the dissection of the subcutaneous tissue and tensor fasciae latae fascia according to the preoperative plan, followed by ligature of blood vessels, exposing and dissecting the joint capsule, and resecting the femoral neck (refer to Fig. 7A). In the PSI group, the patient-specific instrument was used in the greater trochanter region, with the guidance of the PSI, accurate femoral resection level and direction were assured during resection of the femoral head. Subsequently, an experimental stem was inserted after preparation of the femur stem. The coated portion of the stem was inserted into the canal, and the non-coated section was placed above the resection line to achieve the desired insertion depth. Intraoperatively, to ensure a snug fit of the PSI, the designated area’s cartilage and soft tissue were removed in the PSI group, and a single reaming technique was utilized (see Fig. 7B). Following femur preparation, a ceramic liner and trial femoral head of appropriate length were inserted, followed by hip joint reduction and testing. If the intraoperative findings were positive, genuine components were implanted and the joint was realigned accordingly.

Standard perioperative care and patient education were provided to all study participants. Weight-adjusted cefathiamidine and tranexamic acid were administered prior to incision, and ropivacaine was injected into the surgical site and joint area before suturing. Both the PSI and FRH groups followed a standard postoperative rehabilitation program that commenced the day after the surgery.

### Primary outcomes

In this study, postoperative CT radiographic outcomes were evaluated by experienced 4th and 5th-year orthopaedic physicians who conducted standardized radiographic measurements at least 4 weeks after the operation. To avoid bias, the physicians were blinded to the groups and patient information. Each measurement was taken twice, and the mean was used for analysis. The mean absolute error in stem anteversion was determined by computing the absolute discrepancies between the intended and actual anteversion.

### Other outcomes

In this study, the duration from the initial skin incision to wound closure was considered as the surgical operation time. Intraoperative blood loss was recorded using surgical records. The length of hospitalization and time to walking after surgery were also noted. Furthermore, potential complications such as intraoperative fractures, infections, dislocations, and deep venous thrombosis were closely monitored during hospitalization and subsequent follow-up visits.

### Date analyses

Statistical analysis was performed using SPSS version 26 (IBM, Armonk, NY) and GraphPad Prism version 8 (La Jolla, CA). Intraclass correlation coefficient (ICC) was used to compare the variability among interobserver. Accuracy of the procedure was assessed by calculating the absolute error, which represents the difference between the planned and postoperative radiographic measurements. Chi-squared test was used to evaluate discontinuous variables such as incidence and rate between groups. Intergroup differences in continuous variables were assessed using mean and range, and Student t-tests were conducted to compare intergroup differences. Statistical significance was considered when *p* < 0.05.

## Data Availability

All the data used and/or analyzed during the current study are available from Binghua Wu upon reasonable request.

## Abbreviations

PSI: Patient-specific instrument
THA: Total hip arthroplasty
LLD: Leg length discrepancy
BMI: Body mass index
FRH: Free-hand group
TTSW: Time to start walking on the ground after surgery
